# Nanocomposite Electrospun Nanofiber Membranes for Environmental Remediation

**DOI:** 10.3390/ma7021017

**Published:** 2014-02-10

**Authors:** Shahin Homaeigohar, Mady Elbahri

**Affiliations:** 1 Helmholtz-Zentrum Geesthacht, Institute of Polymer Research, Nanochemistry and Nanoengineering, Max-Planck-Str.1, 21502 Geesthacht, Germany; 2 Nanochemistry and Nanoengineering, Institute for Materials Science, Faculty of Engineering, Christian-Albrechts-Universität zu Kiel, Kaiserstrasse 2, 24143 Kiel, Germany

**Keywords:** nanofiber, nanocomposite, nanohybrid, membrane, water filtration, air filtration

## Abstract

Rapid worldwide industrialization and population growth is going to lead to an extensive environmental pollution. Therefore, so many people are currently suffering from the water shortage induced by the respective pollution, as well as poor air quality and a huge fund is wasted in the world each year due to the relevant problems. Environmental remediation necessitates implementation of novel materials and technologies, which are cost and energy efficient. Nanomaterials, with their unique chemical and physical properties, are an optimum solution. Accordingly, there is a strong motivation in seeking nano-based approaches for alleviation of environmental problems in an energy efficient, thereby, inexpensive manner. Thanks to a high porosity and surface area presenting an extraordinary permeability (thereby an energy efficiency) and selectivity, respectively, nanofibrous membranes are a desirable candidate. Their functionality and applicability is even promoted when adopting a nanocomposite strategy. In this case, specific nanofillers, such as metal oxides, carbon nanotubes, precious metals, and smart biological agents, are incorporated either during electrospinning or in the post-processing. Moreover, to meet operational requirements, e.g., to enhance mechanical stability, decrease of pressure drop, *etc.*, nanofibrous membranes are backed by a microfibrous non-woven forming a hybrid membrane. The novel generation of nanocomposite/hybrid nanofibrous membranes can perform extraordinarily well in environmental remediation and control. This reality justifies authoring of this review paper.

## 1. Introduction

**Figure 1.**
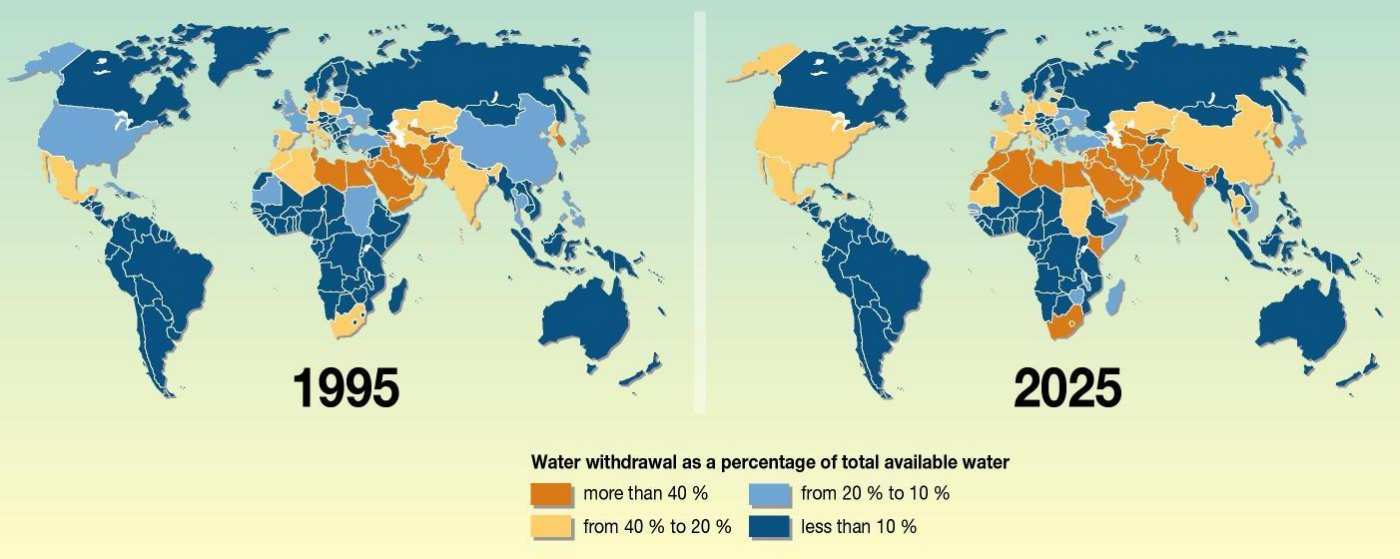
Water scarcity as a global challenge (more than 50% of the world countries will experience water scarcity by 2025) [2].

While for thousands of years of human history, water has been a plentiful resource all around the world and almost a free good, nowadays, the situation is going to be reversed. Unfortunately, water scarcity (Figure 1) is gradually becoming the single greatest threat to food security, human health, and natural ecosystems [1].

Water scarcity is mainly caused by the urban and industrial pollutions. The discharge of wastewater from commercial and industrial wastes, untreated domestic sewage, and chemical contaminants into surface waters leads to a catastrophe. As an example, pollutants, such as arsenic in drinking water, cause bladder, lung, kidney, liver, and skin cancer. Accordingly, 1.2 billion people can hardly obtain safe drinking water, 2.6 billion have little or no sanitation, and every year millions of people die from diseases transmitted through unhealthy water or human excreta [3–5].

**Figure 2.**
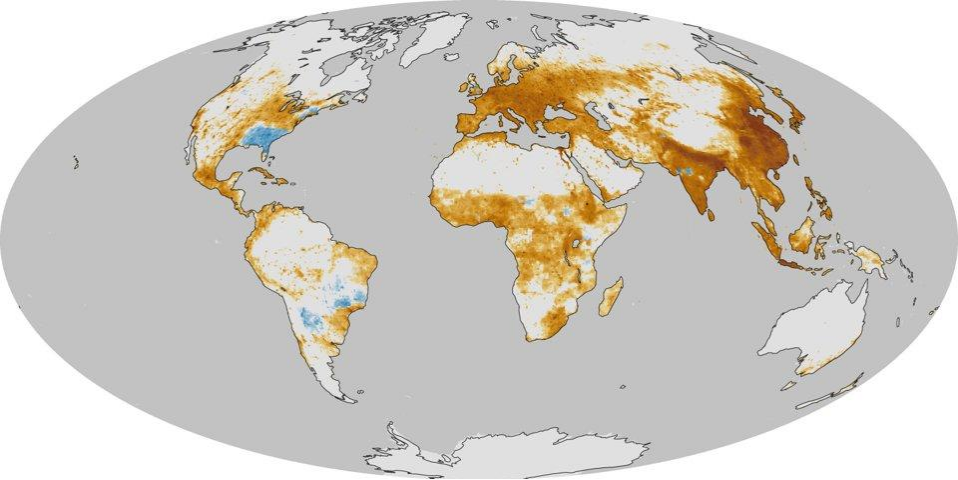
The map presented by the National Aeronautics and Space Administration (NASA) shows the air pollution related mortality rates worldwide (in contrary to the dark brown areas, the blue areas are those where the air quality has been improved and the related death rate has declined) [7].

Introduction of chemicals, particulates, or biological materials into atmosphere via different sources can bring about discomfort, disease, or death to humans (Figure 2). As an example, tiny particles (<2.5 μm) suspending in air are a major cause of cardiovascular and respiratory illnesses [6]. Additionally, the presence of sulfur dioxide, ozone, and nitrogen dioxide is harmful especially for people with asthma. Such pollutants also stimulate allergic reactions.

The progressive air and water pollutions are undoubtedly recognized threats to the world’s environment. Therefore, attention must be switched, as quickly as possible, towards addressing them.

In this regard, global research should be directed towards advanced technologies able to create a clean environment. Filtration technology is one of such leading approaches for making a healthier environment and overcoming the current challenges. For instance, tapping alternative sources of water, such as seawater, rainwater, *etc.*, and removal of available contaminants through a filtration process can be a potential solution for the problem of water shortage.

In addition to the need to a clean environment for a healthier life, filtration is also a necessity for industry. The control over air/waterborn pollutants, hazardous biological agents, as well as allergens, is of the main requirements of industrial companies in food, pharmaceuticals, biotechnology, and semiconductor business [8]. Release of functional nanoparticles (e.g., fullerene, carbon nanotubes, metals, and semiconductors) into the environment is challenging. This issue has newly found a higher importance due to a large scale manufacturing with economically viable processes [6]. According to numerous studies, some of these nanoparticles, e.g., metal and metal oxide nanoparticles and carbon nanotubes are cytotoxic and induce granulomas in animal lungs [9–11].

Overall, an efficient filtration technology for environmental remediation also control of the industrial pollutants is currently a critical need. Hence, there is no wonder to see a market of up to US $700 billion by 2020 for filtration technology [12,13].

Energy efficiency of an environmental technology is of a high importance. This issue stems from the direct correlation of the world increasing population hence energy consumption and the limited available energy sources. The world population is expected to double by the middle of the 21st century. Accordingly global demand for energy services would rise by as much as an order of magnitude by 2050 [14,15]. This fact is interpreted as an increasing concern regarding energy resources and the respective costs. In addition, the energy industry-related environmental problems would be an extra challenge.

The necessity of employing breakthrough filtration technology in an energy and cost efficient way has led to increasing attention in nanostructured membranes, especially nanofibrous ones made via “Electrospinning”.

## 2. Electrospinning

Electrospinning is the most suitable technique for production of nanofibers. The advantages include its relative ease, low cost, high speed, vast materials selection, and versatility. Additionally, the technique allows control over fiber diameter, microstructure, and arrangement [16–18].

**Figure 3.**
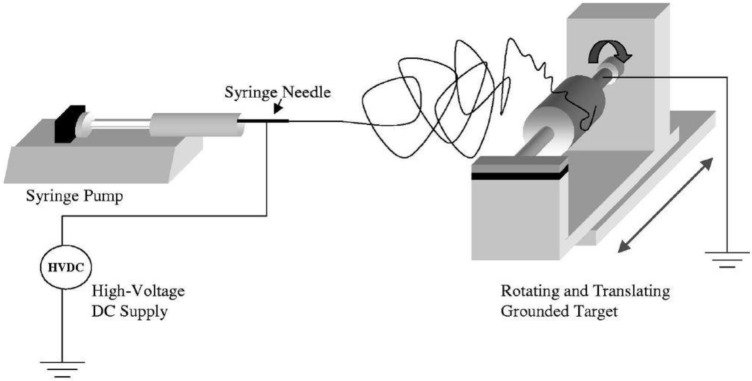
A schematic of the electrospinning process of polymer nanofibers [22].

As shown in Figure 3, this technique is based on three main components: a high voltage supply, a capillary tube containing polymer solution/melt attached to a needle of small diameter, and a metallic collector. To create an electrically charged jet of polymer solution/melt out of the needle, a high voltage is applied between two electrodes connected to the spinning solution/melt and to the collector (normally grounded). The electric field at the tip of the needle electrifies the surface of the droplet of the polymer solution situated on it. Repulsion between charges present at the surface, as well as their attraction to the opposite electrode, induce a force that overcomes the surface tension. As a result, a charged jet is ejected from the tip of the droplet. Due to the mutually repulsive forces of the electric charges in the jets, the polymer solution jet undergoes a bending instability, thereby an elongation and thinning process. Meanwhile, evaporation of the solvent leads to the formation of a charged polymer nanofiber, collected as an interconnected web on the collector [17–21]. The resulting web is composed of randomly aligned nanofibers resembling a non-woven material and a membrane.

The electrospun nanofibrous membranes (ENMs) possess a high porosity and an interconnected porous structure with the pores as big as a few times to a few ten times the fiber diameter. The high porosity implies a higher permeability to fluid streams and the interconnected pores can withstand fouling better. These characteristics bring about low energy consumption. Furthermore, not only the small pore size, but also the huge available surface area, flexibility in surface functionalities, and design of the nanofibrous membranes optimize their adsorptive nature and selectivity [6,18,19,23–27].

The possibility of combining a variety of polymers, particulate nanofillers, and biological agents through electrospinning leads to development of nanocomposite/hybrid nanofibrous membranes with a more optimum filtration efficiency and a much broader domain of environmental applications than their neat counterparts. Such an achievement in the review of electrospinning by Dzenis [21] was mentioned as one of the biggest breakthroughs in the field.

**Figure 4.**
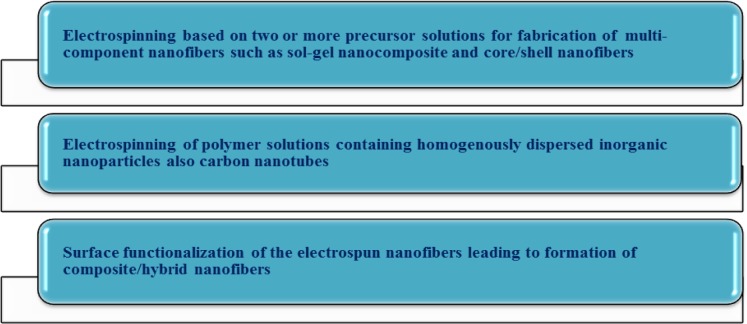
Different fabrication methods of nanocomposite/hybrid ENMs [29–35].

**Figure 5.**
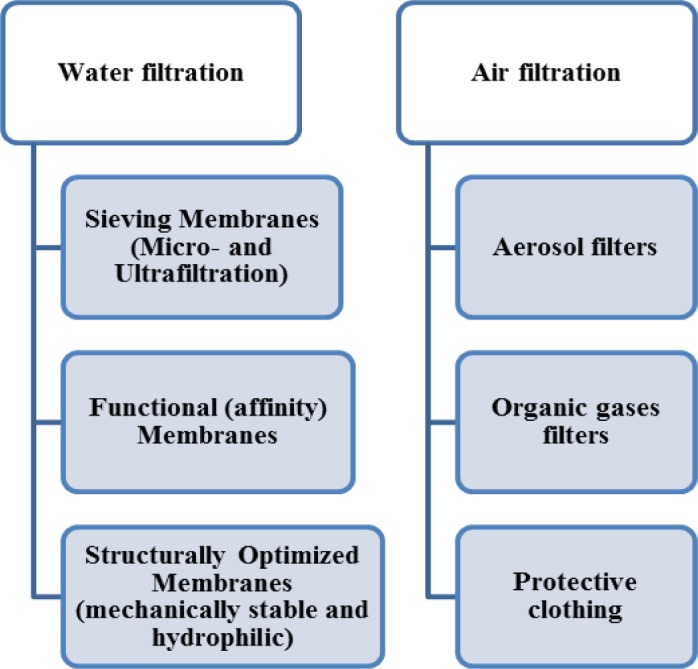
Different classes of nanocomposite/hybrid ENMs based on their environmental applications and performance.

As seen in Figure 4, the nanocomposite synthesis could be conventionally done through blending of insoluble nanoparticles and the polymer solution to be electrospun. In such a manner, the nanoparticles are encapsulated in the solidified nanofibers. Soluble drugs, bacterial agents, and metal oxide sol-gel solutions can be also added to the polymer and electrospun into nonwoven mats [19]. A combination of various moieties different in nature offers the benefits of individual components together. As an example, an inorganic-organic nanocomposite profits from the lightweight, flexibility, and moldability of organic polymers in addition to high strength, chemical resistance, and thermal stability of inorganic additives. Such a promising mixture nominates nanocomposite ENMs for a diverse range of environmental applications including catalytic, micro, and ultrafiltration membranes [28]. Based on our exploring of the literature, as illustrated in Figure 5, the nanocomposite/hybrid ENMs can be categorized depending on the application domain also performance mode.

## 3. Nanocomposite ENMs for Water Filtration

For water treatment, nanofibrous membranes have a quite short history, and, obviously, have not found any real applications [16,36]. This is in contrast to the air filtration area in which the polymeric ENMs have been being used commercially since a few decades ago. One main reason for the lack of interest to research in this area could be inability of the ENMs to withstand high pressures commonly used in water treatment.

Based on the performance mode, the water filtration membranes are divided as sieving and functionalized (affinity) membranes. Membranes such as micro-, ultra- and nanofiltration (MF, UF and NF respectively) ones are frequently used for remediation of wastewaters. The principal separation function is based on sieving mechanism, *i.e.*, needing to a pore with a dimension smaller than the pollutant size. On the other hand, functionalized membranes allow the separation of molecules based on physical/chemical properties or biological functions rather than molecular weight/size. This interaction-based separation circumvents any need to design of very small pore sizes thereby huge feed pressures. Indeed a functionalized membrane of which the surface is equipped to immobilized specific ligands, separates or captures molecules selectively [37,38]. Such a classification also applies to the ENMs under investigation.

### 3.1. Sieving Membranes

Depending on the pore size and filtration application, the water purification process can be defined as microfiltration, ultrafiltration, nanofiltration, reverse osmosis (RO), and forward osmosis (FO). Thanks to remarkable water permeability, ENMs have been mostly explored for MF and UF applications [39–41]. However, to provide water channels in the barrier layer thereby minimizing fouling, they could be considered for NF application as well [36,42].

#### 3.1.1. Nanocomposite ENMs for Microfiltration

According to the Baker’s definition: “Microfiltration refers to filtration processes that use porous membranes to separate suspended particles with diameters between 0.1 and 10 μm” [43]. Pre-filtration of waste water streams is a known application of the MF membranes. Pre-filters are used to remove coarse particles thereby maintaining the performance of the down stream filter unit such as RO and NF membranes for much longer time before cleaning and/or replacement [44,45]. The rejection of micron scale unwanted species of, e.g., particles, flocs, bacteria, *etc.*, is usually done by MF membranes. In fact, two main objectives of water filtration including purification and disinfection are simultaneously met. Conventional MF membranes, however, suffer from low flux and high fouling tendency. These drawbacks are mainly due to their geometrical structure of pores, the corresponding pore size distribution [46], and undesirable macro-void formation across the whole membrane thickness [47]. Free of these disadvantages, the ENMs could be good candidates for liquid microfiltration. These membranes possess a pore size distribution from sub-micron levels to a few micrometers in the range of MF membranes. In addition, compared to conventional phase inverted membranes, they contain a higher porosity, hence, permeability.

Despite the advantages of ENMs for filtration, as mentioned earlier, they are mechanically weak and under pressure undergo pore collapse. In addition, accumulation of electrostatic charges during electrospinning is a problem, which challenges the handling of ENMs. Spinning the nanofibers directly on a stronger support thereby forming a hybrid ENM is one way of alleviating the handling issue also pore collapse [39,44,48].

**Figure 6.**
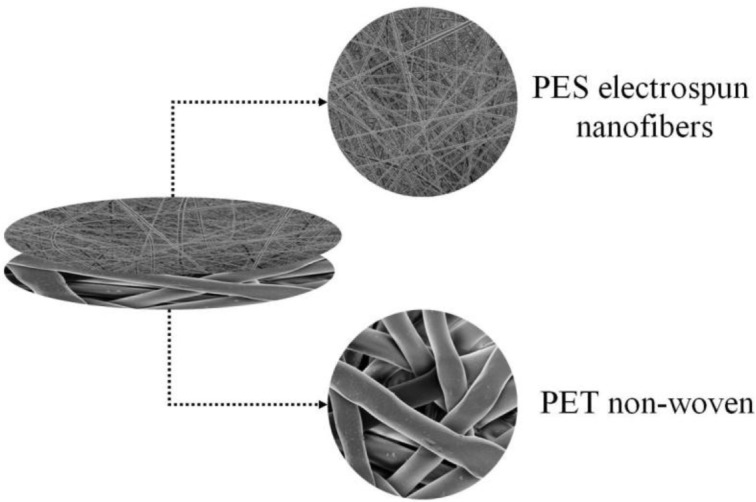
PES/PET hybrid ENM [44].

The filtration potential of a hybrid ENM was recently investigated by Homaeigohar *et al.* [44]. In this study, polyethersulfone (PES) nanofibers (260 ± 110 nm in diameter) were deposited on a technical microfibrous non-woven of poly(ethylene terephthalate) (PET). This hybridization was done to alleviate difficult handling of the nanofibrous web also to provide mechanical strength (Figure 6). This hybrid ENM was studied as a pre-filter in removal of particles from water. To enhance the interfacial stability, a heat treatment was also performed.

Retention tests with polystyrene suspensions demonstrated that the filtration performance of the hybrid ENM strongly depends on size distribution of the suspended particles. When the microparticles are present in the feed, the major rejection of the particles is performed within the first hour of the measurement. The flux is very high and pressure drop very low and almost negligible. In the case of a feed containing only submicron particles, the major rejection is accomplished within the first hour. However, due to pore blocking, the flux declines and the pressure drop rises drastically [44]. In fact, for big particles, the ENM acts as a screen filter, easily washable, while for smaller particles as a depth filter, forming a cake layer and significantly fouled [6,40]. This research demonstrates the filtration potential of the PES/PET hybrid ENM for pre-treatment of water. The ENM is able to efficiently remove micron scale particles from water with very high permeability, *i.e.*, low energy consumption.

**Figure 7.**
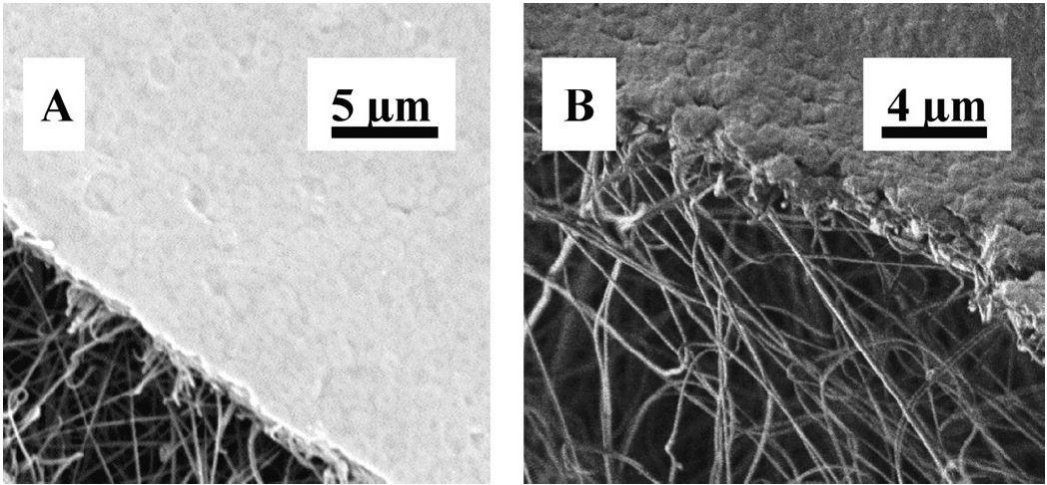
SEM images showing (**A**) surface and (**B**) cross-section views of PAN/PET hybrid ENM after *E. coli* suspension filtration [49].

Similarly, Wang *et al.* [49] studied the ability of a hybrid polyacrylonitrile (PAN)/PET ENM for microfiltration. Excluding the nonwoven support layer, the membrane’s characteristics included: a thickness of 200 ± 10 μm with a mean fiber diameter of 100 ± 20 nm, a maximum pore size of 0.62 ± 0.03 μm, and a mean pore size of 0.22 ± 0.01 μm. In terms of permeability, this hybrid ENM exhibited a two to three times higher flux compared to the commercial counterparts with the same mean pore size. Moreover, the membrane could reject microparticles, also bacteria (LRV = 6) (Figure 7), substantially. Accordingly a high flux hybrid ENM has been proposed for a MF purpose.

#### 3.1.2. Nanocomposite ENMs for Ultrafiltration

Ultrafiltration (UF) is another liquid filtration process discriminating a diverse range of pollutants, such as viruses, emulsions, proteins and colloids as big as about 1–100 nm [43]. ENMs can be also used as an UF membrane in case of having a surface pore size less than 0.1μm. Nevertheless, with such a pore size and high surface area to volume ratio, ENMs are very prone to a rapid fouling with significant loss of flux. Unless, they are used with a thin film as coating, *i.e.*, as a thin film composite (TFC) membrane [6]. This UF TFC membrane consists of a nonwoven microfibrous substrate, an electrospun nanofibrous mid-layer, and a barrier layer (fabricated by coating or interfacial polymerization). The highly porous electrospun nanofibrous mid-layer creates a high permeability. Hence, as compared to the conventional TFC membranes with an asymmetric porous phase inverted mid-layer, an enhanced filtration efficiency is expected [6].

**Figure 8.**
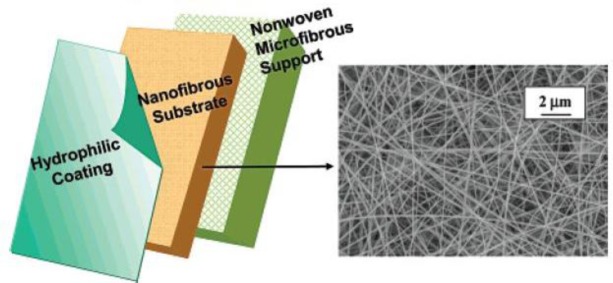
Schematic structure of the UF TFC electropun nanofibrous membrane (left) and representative SEM image of electrospun PVA midlayer (right) [50].

Some studies have proved the efficiency of such a hybrid membrane. For example, as presented in Figure 8, Wang *et al.* [50] used a cross-linked polyvinyl alcohol (PVA) electrospun nanofibrous scaffold as the mid-layer in such a TFC membrane. The nanofibers had a diameter of 130 to 300 nm and porosity (bulk and surface) of the scaffold was 83%. As the hydrophilic barrier top layer, both cross-linked PVA hydrogel and PEBAX^®^ 1074 (a polyamide–polyethylene glycol copolymer, Atofina, philadelphia, PA, USA) were employed. To evaluate this nanofibrous TFC membrane, a model suspension of bilge water, containing soybean oil (1350 ppm) and DC 193 (polysiloxane–polyethylene glycol) emulsifier (150 ppm) was used. The results showed a considerably higher flux rate for the nanofibrous TFC compared to conventional TFCs having the same barrier layer. The permeate flux and rejection rate were strongly dependent to the cross-linking density of the PVA barrier layer. This parameter was controllable by the amount of glutaraldehyde as the cross linker. Moreover, when oxidized multi-wall carbon nanotubes (MWNTs) (up to 10 wt% of the polymer weight) were added to the barrier layer, the permeate flux enhanced up to three times for PEBAX^®^ and five times for cross-linked PVA (while maintaining the same high rejection ratio around 99.8%). The increase of the flux in the TFC nanofibrous membranes with MWNT incorporated barrier layer was more than 10-fold of that in a typical UF TFC membrane. This significant increase of flux could be due to the new water channels exposed on the surface of oxidized carbon nanotubes [50].

Similarly, Yoon *et al.* [51] made a new type of high flux UF/NF membrane based on an ENM. The nanofibrous membrane contained PAN nanofibers coated by a thin, hydrophilic, water-resistant, but water-permeable chitosan top layer. Possessing a porous (~0%) scaffold made of PAN nanofibers (124–720 nm in diameter) coated by a 1 μm thick chitosan top layer, the hybrid membrane showed a flux rate that was an order of magnitude higher than that of commercial NF membranes in 24 h operation. This permeability was with no compromise of rejection efficiency (>99.9%) for oily waste-water filtration.

### 3.2. Affinity Membranes

In contrast to the size excluding membranes, affinity membranes discriminate molecules based on physical/chemical properties or biological functions. Relying on the specific ligands immobilized or secondary phases present at the surface, the nanofibers capture and separate molecules selectively [38]. Accordingly, substances such as nanoparticles, ions, organics, bacteria, *etc.*, are removed from waste-water streams in a customized manner.

#### 3.2.1. Nanocomposite ENMs for Nanofluid Filtration

In terms of selectivity, the ENMs usually possess a pore size in the range of microfiltration (0.1–10 μm). Therefore, they are hardly able to catch particles below this range [39,40,44,45,52–54]. To broaden the range of applicability to removal of nanosubstances, ENMs should be equipped to the other separation mechanism, *i.e.*, adsorption as well.

**Figure 9.**
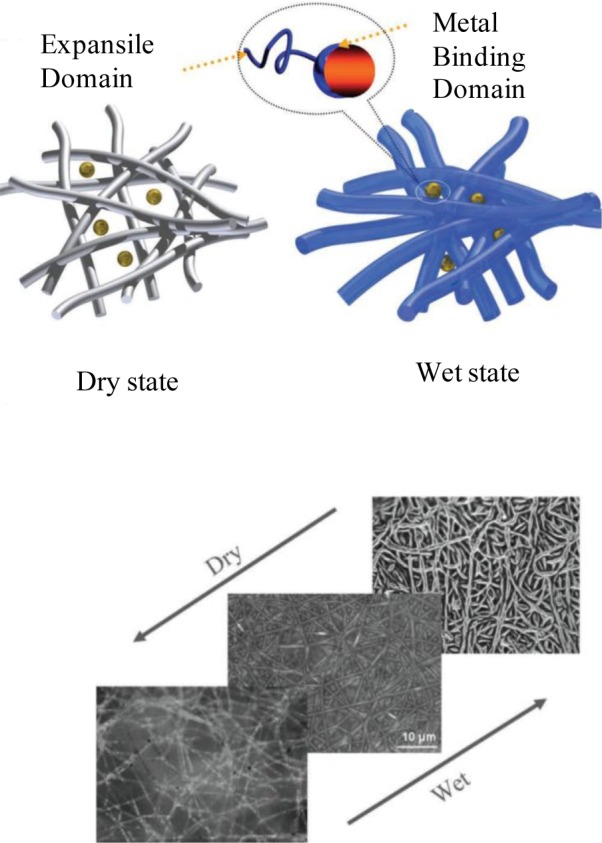
Capturing of metal nanoparticles through the wetting induced-conformational change of the proteins immobilized onto nanofibers [26].

Recently, we showed a macroporous bionanohybrid membrane consisting of polymeric nanofibers and proteins [26]. In the same manner as a “carnivorous plant” hunts its prey, this membrane was able to filter out tiny nano-scaled particles present in aqueous solutions. In this study, the nanofibers made of poly(acrylonitrile-co-glycidyl methacrylate) (PANGMA) (100 nm in diameter) were surface functionalized via protein [Bovine Serum Albumin (BSA)] immobilization. Due to biofunctionalization, the nanofibers were slightly enlarged in diameter to 126 nm. In this bionanohybrid ENM, upon wetting during filtration and increase of pH to above isoelectric point of the protein, the protein undergoes a conformational change. This transformation leads to emergence of the previously hidden functional groups. The emerged functional moieties are able to bind to water molecules (thereby protein swelling) and nanosolids to be filtered. Moreover, the swollen protein makes a higher steric hindrance facilitating the capturing of the nanosolids. In this carnivorous plant-like membrane, the swollen protein immobilized nanofibers act as the spread leaves of the plant wherein the emerged functional groups catching the nanoparticles as the sticky mucilage (Figure 9).

**Figure 10.**
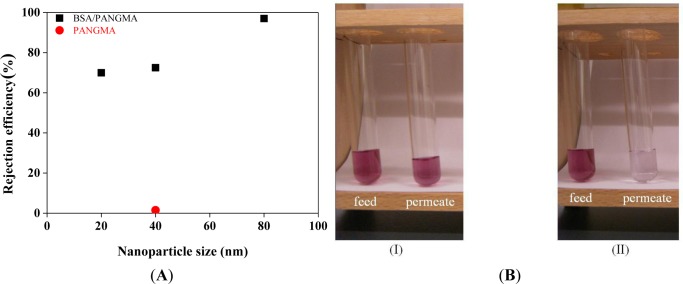
(**A**) Extraordinary retention efficiency of the BSA/PANGMA ENMs for Au nanoparticles and (**B**) visual comparisons between the feed and permeated samples through the neat (I) and BSA/PANGMA ENMs (II).

Retention ability of the BSA/PANGMA ENMs for Au nanoparticles is demonstrated in Figure 10A. While the neat PANGMA ENM is able to reject a negligible part (1.5%) of the 40 nm Au nanoparticles, the biofunctionalized ENM’s rejection efficiency is 72.5%.

Visual inspection of the feed and permeates clearly reveals the high retention ability of the BSA/PANGMA ENM. As seen in Figure 10B, color intensity of the feed sample drastically decreases after permeation through the biofunctionalized ENM. The retention efficiency for 80 nm Au nanoparticles is even more as high as 97%, whereas this value for the 20 nm nanoparticles reduces to 70%, still high and promising. In addition to such an amazing retention efficiency, the permeate flux recorded when filtration of 80 nm Au nanosuspension, was also as considerable as 9000 l/h.m^2^. This permeability is much higher than that reported for conventional MF/UF membranes [53]. Noteworthy, there was no major feed pressure and the experiment was performed under the atmospheric pressure, *i.e.*, a very low energy consumption.

In our recent publications, we showed how the protein functionalization modifies also the physicochemical properties of the ENM [55]. In addition, the bionanohybrid demonstrated an ability in capturing even trace amounts of protein and enzymes from wastewater streams [37]. This performance will be further elaborated in a next section.

#### 3.2.2. Nanocomposite ENMs for Filtration of Heavy Metals

In aquatic systems, heavy metals such as copper, cadmium, and chromium show a high toxicity and easily accumulate in living organisms. Accordingly, as a serious health problem, they should be strictly regulated in water in the level of around a few tenths of ppb or less [6,52].

Two conventional methods for elimination of such pollutants are adsorption and filtration. Interestingly, ENMs with a tunable small pore size and high surface area are able to offer both techniques [52]. For example, silk fibroin and a blend of silk fibroin with wool keratose ENMs have shown high removal efficiency for heavy metals as compared to conventional materials, such as wool silver and filter paper [56].

In a research by Hota *et al.* [16,57], a nanocomposite ENM was developed by incorporation of nano-boehmite into nylon-6 and polycaprolactone (PCL) electrospun nanofibers. According to SEM micrographs, the fiber diameters were found to be in the range of 300–600 nm for nylon 6 and 400–850 nm for nylon-boehmite, 0.9–1.2 μm for PCL and 1.0–1.5 μm for PCL-boehmite composite fibers. This membrane could show a Cd^2+^ removal ability of 0.2 μg/mg. Such a binding capacity of the cadmium ions was due to the high surface area of the nanocomposite nanofibers. However, because of encapsulation of the functional nanoparticles within the nanofibers and less exposure to the heavy metal ions, the removal efficiency was not comparable with that of the nanofibers containing functional polymeric molecules.

In another study by Xiao *et al.* [58] a nanocomposite ENM, based on polyacrylic acid (PAA)/polyvinyl alcohol (PVA) nanofibers containing MWNTs, was fabricated. The nanocomposite nanofibers were further surface decorated with zero-valent iron (ZVI) nanoparticles. While MWNTs were incorporated into the nanofibers to optimize their mechanical stability, ZVI nanoparticles were added to interact and remove Cu^2+^ ions from water. According to the results, the Cu^2+^ chemisorption process occurs via chemical reduction of the ions and their subsequent deposition on surface of the ZVI nanoparticles. Thanks to a huge specific surface area, the uniformly dispersed ZVI nanoparticles of the nanocomposite ENM could effectively capture copper ions. Therefore, this nanocomposite membrane has a promising potential for removal of heavy metal ions and an efficient water treatment.

#### 3.2.3. Nanocomposite ENMs for Filtration of Organic Materials

Of the most important pollutants, water quality is highly sensitive to the organic ones, such as oil, protein, humic acid, *etc.* Such organics, even at a very low concentration, e.g., no more than 1% of the pollution in a river, are able to use up the dissolved oxygen, making the water free of any life [59].

While proteins are of the main pollutants usually removed by UF membranes, a functionalized ENM could be an attractive substitute. The interconnected porosity of an ENM gives rise to an extraordinary permeability, thereby, very low energy consumption. Moreover, its huge surface area enables a high amount of functionalization required for greatly selective membrane applications. Accordingly, the functionalized polyurethane, polysulfone, polyacrylonitrile, and cellulose ENMs have shown a promising potential for protein (e.g., IgG, BSA, lipase, bromelain, *etc.*) separations [38,52,60–62].

As mentioned earlier, recently, our group biofunctionalized PANGMA ENMs via immobilization of BSA protein onto the nanofibers [37]. This novel bionanohybrid membrane, despite its macroporous structure, is able to efficiently (~0%) separate even trace amounts (as low as 2 mg/L) of protein and enzyme (BSA and *Candida antarctica Lipase* B (Cal-B), respectively) from water. Such an extraordinary protein selectivity at this level of pollutant concentration for a nanofibrous membrane has never been reported. In spite of a negligible non-specific adsorption of both BSA and Cal-B to the PANGMA ENM (8%), the separation efficiency of the biofunctionalized membrane for this biomolecules reaches to 88% and 81%, respectively. The optimum separation efficiency is due to the water-induced conformational change of the biofunctional agent. The conformational change not only exposes more functional groups available to catch the biomolecules but also leads to swelling of the nanofibers thereby a higher steric hindrance for the solutes.

A capturing mechanism of organics will be perfected by a dissociation process. Organic pollutants, such as dyes and pesticides, are readily decomposed in aqueous solutions by photocatalysis at the presence of semiconductor metal oxides such as titanium oxide (TiO_2_), zinc oxide (ZnO), tin oxide (SnO_2_), and copper oxide (CuO). These materials due to their size-tunable physicochemical properties, high activities, durability, and low cost have been widely investigated in photocatalytic degradation of organic pollutants. In this regard, a major challenge is the fast recombination of photo-generated electron/hole pairs in the bulk semiconductors thereby lowering their photo-catalytic efficiency [29,63]. Composite materials by virtue of the different Fermi levels of the constituents could be a hindering solution. In addition, addition of nanoparticulate semiconductors to nanofibers, while maintaining their exposure at a high surface area, eases their recycling and prevents their aggregation.

**Figure 11.**
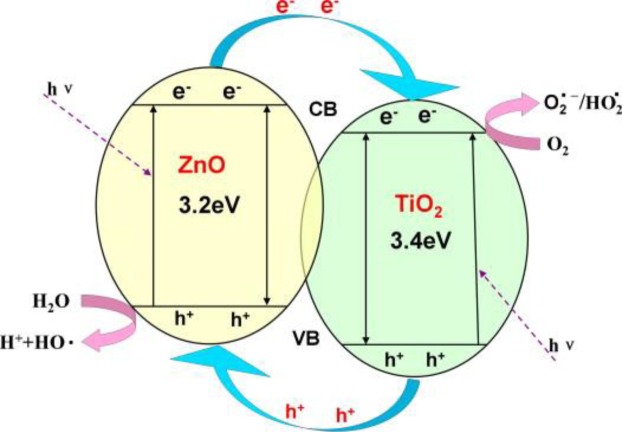
The mechanism of charge separation and photocatalytic activity of the TiO_2_/ZnO nanofibers [64].

Among the photocatalytic materials, TiO_2_ is a well-known one being used extensively. This semiconductor can photocatalytically oxidize toxic substances into nontoxic ones of carbon dioxide and water. Accordingly, it has been widely used for fabricating composite nanofibers. As an example, a membrane composed of TiO_2_/ZnO composite nanofibers (85–200 nm in diameter) possessing ZnTiO_3_, ZnO, and TiO_2_ crystallites [64] has shown a decomposition capacity of nearly 100% for Rhodamine-B (RhB) and 85% for phenol. Addition of ZnO (optimally 15.76 wt%) in the composite nanofibers is a factor improving the photocatalytic efficiency. This behavior could be due to a slower charge recombination on the TiO_2_/ZnO nanofiber thereby a higher degradation rate (Figure 11).

Kim *et al.* [65] fabricated TiO_2_/poly(dimethyl siloxane) (PDMS) composite fibrous membranes for photocatalytic degradation of methylene blue. While the mechanical stability of the membrane is improved by addition of PDMS fibers (11.4 μm in diameter), their photo-degradation ability is fully dependent on the content of TiO_2_ fibers (1.11 μm in diameter). In another study by Nakata *et al.* [66], the researchers made Al_2_O_3_/TiO_2_ composite fibers (1.8–3.4 μm in diameter) with an optimum performance in UV induced photocatalytic decomposition of acetaldehyde.

The large band gap of TiO_2_ is a big barrier ahead of its wide use in decontamination of waste water streams. To activate photogeneration of electron/hole pairs in the conduction and valence bands, respectively, UV light should be applied to supply the energy equal to or higher than that of the corresponding band gap of TiO_2_. As UV light constitutes only 5% of the Sun’s energy reaching to the Earth’s surface, a wide research is in progress to shift the optical response of TiO_2_ from UV to the visible spectral range [67]. Im *et al.* [67] succeeded to achieve this aim by a multielement doping approach. Subsequently, they immobilized the modified TiO_2_ particles in/on hydrogel fibers (155 ± 25 nm in diameter). This photocatalyst system was used in degradation of dyes in two grades of anionic and cationic under visible light. In this study, the dye was optimally captured by the hydrogel fibers and degraded by the TiO_2_ particles. In another study by this group, TiO_2_ particles were uniformly dispersed on PAN nanofibers (800 nm in diameter) to create a nanohybrid ENM for the UV light induced photo-decomposition of RhB dye [68]. The TiO_2_/PAN ENM suspending on the dye solution showed a high dye removal capacity of 60% in a photocatalytic manner after 48 h.

ZnO is another semiconductor metal oxide extensively studied for photocatalysis of dye pollutants. For example, Ye *et al.* [69] made a nanocomposite ENM through incorporation of ZnO nanocrystallites into cellulose nanofibers (159 ± 28 nm in diameter). The dye (RhB) removal capacity of this system induced by the photocatalytic activity of ZnO was evaluated under sunlight. This nanocomposite ENM benefits from not only the photocatalytic ability of ZnO nanoparticles but also the thermal and mechanical stability and solvent resistance of the cellulose nanofibers. A strong photocatalytic efficiency towards the degradation of RhB was observed, and almost 50% of the dye was decomposed after 24 h visible light irradiation.

#### 3.2.4. Nanocomposite ENMs for Removal of Microorganisms

The pathogenic microorganisms like *Cryptosporidium parvum* and *Giardia lamblia* are so hazardous that their presence in water can cause serious illnesses. For this reason, in many countries, removal of them from water is compulsory.

**Figure 12.**
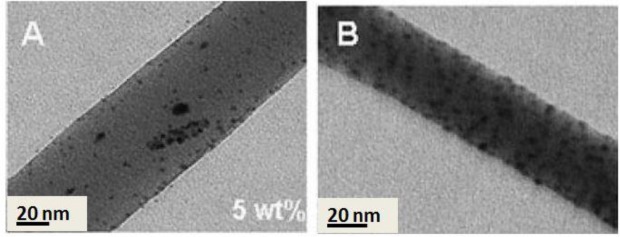
TEM images of cellulose acetate nanofibers containing 5 wt% AgNO_3_ (**A**) before UV irradiation and (**B**) after UV irradiation [70].

Functionalized nanofibrous membranes can be beneficial in disinfection of water [52]. Implementation of substances such as elemental silver and silver salts, silver-TiO_2_ systems, and quaternary ammonium salt-containing cationic polymers can induce good antimicrobial properties to the membranes [16]. By virtue of available high surface area, antimicrobial agents incorporated ENMs can offer a very promising efficiency in removal of such pollutants [6]. One of these able ENMs has been developed by Lala *et al.* [70] (Figure 12). They fabricated several silver impregnated polymeric nanofibrous membranes and evaluated their antimicrobial capability using two gram negative bacterial groups: *E. coli* and *P. aeruginosa*. The results were quite promising in terms of antimicrobial activity of the membranes when incubated with bacteria [52]. In addition to incorporating antimicrobial properties, silver nanoparticles can minimize the formation of biofilm on the surface of membranes hence inducing an antibiofouling effect [16].

Hydrophilic nylon-6 is one of the famous electrospinning membrane materials [45]. Accordingly, Park *et al.* [29,71] developed nylon-6 nanofibers (150–250 nm in diameter) containing well dispersed silver nanoparticles. Such a nanocomposite ENM showed an optimized antibacterial activity as compared to its neat counterpart thereby a high potential as a water filtration membrane.

Pant *et al.* [72] also synthesized a spider-net like composite mat of nylon-6 functionalized with TiO_2_ nanoparticles. The added nanoparticles induced a higher hydrophilicity, mechanical strength, antimicrobial, and UV protecting ability to the nanocomposite ENM. This nanostructured membrane with an improved wettability, also antifouling property, can be a potential candidate for future water filtration applications.

In another study, Chen *et al.* [16,73] fabricated a composite ENM of AgNO_3_/polypyrrole/polyacrylonitrile. They showed that embedding of silver nitrate into the nanofibers (300 nm in diameter) leads to a more optimum electrical conductivity also antimicrobial property. The former feature could be implemented as an indication of fouling of the membrane.

Remembering the ability of our BSA/PANGMA ENM in capturing biomolecules, biofunctionalized nanofibrous membranes could be a promising alternative for the nanocomposite nanofibrous membranes in removal of microorganisms. In this case, thanks to an optimum biocompatibility, their application domain would be extended from environmental remediation to biomedicine. The respective study is in progress in our laboratory.

### 3.3. Durable Nanocomposite ENMs for Pressure Driven Water Filtration

Despite optimum filtration characteristics, ENMs especially in pressure-driven liquid separations, such as MF and UF, are not structurally durable. As shown by us [44], despite an extraordinary permeability, due to the very high porosity and surface area, the nanofibrous membranes are susceptible to mechanical breakdowns. This could be also partly due to improper alignment of the polymer chains during electrospinning and an inadequate interaction between them in nanofibers [36].

An ENM could be employed for filtration in either dead-end or cross flow modes. While in the former mode, the feed liquid is passed through the membrane by pressure, for the latter it is pumped across the membrane parallel to the surface [74]. Depending on the filtration mode, ENMs should be mechanically stable under different kinds of stresses, *i.e.*, shear, compressive, and tensile ones. Accordingly, this topic is under extensive investigations to minimize eventual mechanical failures during water filtration processes.

To enhance the mechanical stability, recently, we benefited from the residual solvent of the electrospun nanofibers to induce an interfiber adhesion through a thermal treatment. This approach was successful in enhancement of the mechanical properties of a PES ENM reflected as a higher elastic modulus and compaction resistance [75].

In a newly started study by our group [23], we aim to investigate the effect of a nanohybrid structure, so-called “nano galaxy”, composed of nanofibers, beads and spherical solidified droplets on the overall mechanical performance of a nanofibrous membrane in water treatment. The beads (and eventually spheres) are assumed to provide anchorage for load transfer as they have shown in nanocomposite structures [16]. This nanohybrid structure is formed via electro-spinning/spraying of a dilute polymer solution. Indeed, an unstable charged jet of the solution creates a hybrid of nanofibers, beads and spheres.

**Figure 13.**
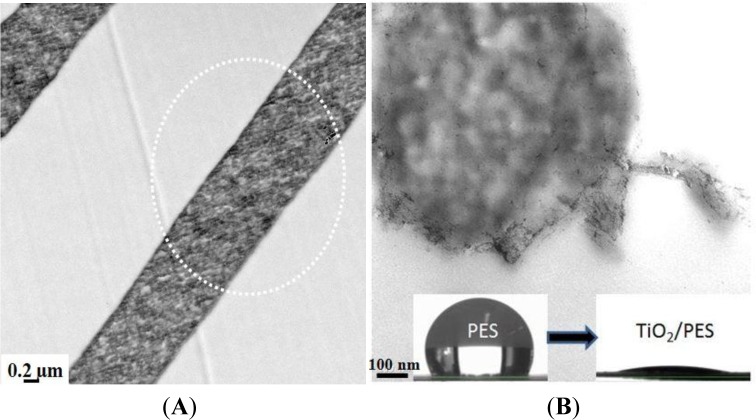
TEM observation of (**A**) ZrO_2_/PES fibers [24] and (**B**) TiO_2_/PES nanofibers (adjacent to a bead with the same composition) (the inset picture implies the superhydrophilicity effect of the composite nanofibrous membrane) [24,25].

Incorporation of nanoparticles into polymeric materials enhances the mechanical properties of the composites. As an example, for nearly a century, nanoparticular fillers (e.g., carbon black) have been used in the rubber industry to increase the mechanical strength of rubber composites [76]. This idea can be also applicable for strengthening the electrospun nanofibers used as filtration membranes. A nanocomposite strategy, as proved by us and other researchers, fulfills the requirement of mechanical stabilization. In our study, zirconia (ZrO_2_) nanoparticles as a novel nanofiller in the membrane technology were selected to mechanically stabilize PES ENMs [25]. Intentionally, physical blending was employed to incorporate zirconia nanoparticles into the fibers (Figure 13A). Depending on the filler concentration, the nanocomposite fibers were as large as 0.3–1.6 μm in diameter. Embedding of the nanofiller could optimize the mechanical properties of the ENM especially its compaction resistance (up to 15%). Reflected in the water permeability (an increment of 100%–400% depending on the composition and ignoring that of the ZrO_2_/PES electrospun microfibrous membrane), pore collapse was prevented and the porosity was preserved. Such an optimum performance was re-obtained when using TiO_2_ nanoparticles. Through a sol-gel approach, the nanoparticles were grown on/near to the surface of the PES nanofibers (Figure 13B). This nanocomposite ENM exhibited a desirable mechanical performance as well as a superhydrophilicity effect. Therefore, a new concept was adopted, not only to mechanically stabilize the nanofibrous membranes, but also to hydrophilize them [24].

In another study by Lijo *et al.* [77] TiO_2_ nanoparticles were electrosprayed over electrospun polyimide nanofibers (251 ± 37 nm in diameter) thereby forming a nanohybrid ENM. This approach was efficient in optimizing the mechanical properties of the nanohybrid ENM (0.36 MPa for polyimide and 0.65 MPa for polyimide/TiO_2_ membrane).

### 3.4. Hydrophilic Nanocomposite ENMs for Water Filtration

For an ENM, a combination of hydrophobic membrane materials frequently used, a high surface area and roughness bring about a high hydrophobicity and fouling tendency. Therefore, to preserve high permeability and filtration efficiency, ENMs should be hydrophilized.

Inorganic nanoparticles are inherently hydrophilic and as a filler can increase the hydrophilicity of the host polymer matrix. Recently, this group of nanoparticles has been proposed to improve the properties of polymeric membranes including antifouling, permeation, thermal and mechanical properties. The application fields of such nanocomposite membranes encompass MF, UF, gas separation, as well as pervaporation.

TiO_2_ nanoparticles are one of the mostly used inorganic nanoparticles, especially because of their high hydrophilicity, chemical stability, antibacterial property, innocuity, and low cost [78–80]. In one of our studies, to compensate the lack of wettability of PES ENMs, they were chosen as nanofiller [24]. Due to the different polarity status of the polymer matrix and inorganic nanoparticles, one of the major fabrication challenges of such nanocomposite membranes is the uniform dispersion of the filler [81]. Physical blending of inorganic nanoparticles with polymer matrix is not a suitable approach to create a homogenous dispersion. In this case, aggregation is always problematic unless the surface of the nanoparticles is organically hydrophobized [82]. To minimize the aggregation, two different approaches have been proposed [81]:

1. *In situ* polymerization of polymer monomer in the presence of surface-modified inorganic particles [83,84]. Induction of a proper dispersibility and long-term aggregation resistance to the nanoparticles, however, is an obstacle ahead [85].
2. Inversely, the other method called sol-gel is based on *in situ* generation of inorganic particles in organic phase, *i.e.*, bulk polymer, polymer solution and monomer systems [86,87]. In such an approach, owing to *in situ* nucleation and growth of the particles inside the host polymer matrix, they are confined and not able to contact each other and aggregate.

To prepare nanoparticulate composite materials with a uniform dispersion of nanofillers, the second approach seems to be more promising. The advantages include very fine particle size and low aggregation, mainly due to *in situ* growth of inorganic nanoparticles in a limited space of polymer matrix. In our study, the PES electrospun nanofibers were modified using TiO_2_ nanoparticles by a sol-gel process. Depending on the filler concentration, the nanocomposite fibers were as large as 110–280 nm in diameter. The technique assures surface residence of the nanoparticles with least agglomeration (Figure 13B) [24]. The nanocomposite ENMs showed amazing properties in terms of mechanical stability (a higher storage modulus of 10–30 MPa) also wettability (a superhydrophilicity effect, *i.e.*, a water contact angle less than 5; Figure 13B inset picture). The former feature was a result of hydrogen bonding between TiO_2_ and the functional groups of PES, such as sulfone or ether. In the mechanically stable also hydrophilic ENM, porous structure is preserved and water with less resistance passes through the membrane. The resultant performance was bloomed as an incredibly high water permeability, thereby, low energy consumption and a long life span.

Tijing *et al.* [88] developed a superhydrophilic and at the same time antibacterial nanocomposite ENM composed of tourmaline (TM) nanoparticles decorated polyurethane (PU) nanofibers. The nanocomposite ENM (3 wt% TM nanoparticles) exhibited an increase in tensile strength and modulus of 75% and 87%, respectively compared to the neat PU ENM. At the composition of 5 wt% TM/PU ENM, a superhydrophlic surface with a contact angle of 13° was obtained. Through FTIR, it was specified that there is a hydrogen bonding between TM nanoparticles and PU chains. This bonding is assumed to be the cause of optimized mechanical stability of the nanocomposite ENM. In addition to the enhanced physicochemical properties, bacterial tests also confirmed the high antibacterial activity of the TM/PU ENMs against *Escherichia coli* (Gram-negative) and *Enterococci* (Gram-positive). A promising combination of improved mechanical stability, superhydrophilicity and antibacterial properties of the nanocomposite ENM proves its suitability for water filtration applications.

## 4. Nanocomposite ENMs for Air Filtration

The efficient air filters are nowadays needed more than ever. The reasons could be the progressive trend of air pollution especially in metropolitans and the necessity of removal of hazardous particles from work environments, e.g., in semiconductor and the health-care industry. Moreover, for specialized applications such as protection against toxic gaseous agents in the air, filtration is of a high importance.

### 4.1. Aerosol Filtration

**Figure 14.**
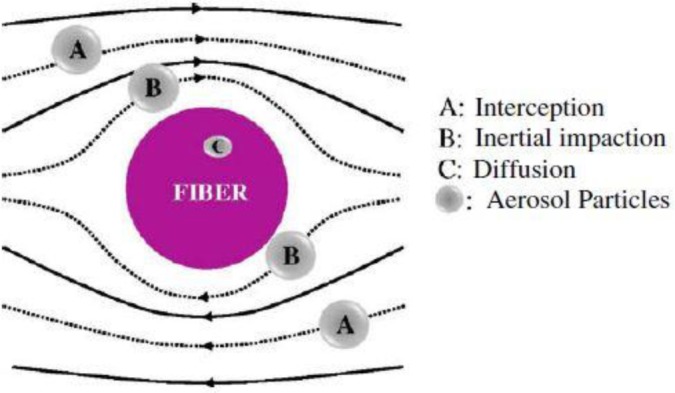
The main aerosol capturing mechanisms of a fibrous filter [89].

Air filtration is conventionally performed by fibrous filters primarily due to their high collection efficiency also durability. As shown in Figure 14, aerosol particles could be deposited on and captured by a fiber through five different mechanisms including: interception, inertial impaction, diffusion, gravitational force, and electrostatic attraction. Usually filtration of particles is done as a result of combination of all these mechanisms with different contributions. For instance, for most particle sizes, the gravitational force has the least role in filtration. To capture coarse particles, Brownian diffusion has an ignorable role while it is the dominant filtration mechanism for fine particles. For the submicron particles, the most important capturing mechanisms include Brownian diffusion, interception, and inertial impaction, as well as the electrostatic attraction [29].

Compared to conventional filtration microfibers, nanofibers possess a much smaller diameter thereby offering a higher chance of inertial impaction and interception, *i.e.*, a more optimum filtration efficiency. Moreover, by virtue of slip flow at the nanofiber surface (for the nanofibers with diameter smaller than 500 nm), drag force on the fiber and consequently pressure drop decreases. Slip flow also results in passing more contaminants near the surface of the nanofibers, hence, the inertial impaction and interception efficiencies rise. As a result, filtration capability of the nanofibrous membrane increases for the same pressure drop as compared with conventional fiber mats. Additionally, the very high surface area of the functionalized nanofibers facilitates adsorption of contaminants from air. All these desirable features have increased attentions to nanofibrous membranes for air filtration applications [52]. The first nanofibrous membranes for air filtration were employed in the early 1980s and, since then, further developed up to now.

An outstanding air filter should provide a high filtration capacity along with a low-pressure drop. Nanofiber mats are able to offer such advantages simultaneously. Kim *et al.* [29,90] developed a hybrid air filter based on PAN nanofibers mounted on a commercial melt-blown poly(propylene) (PP) nonwoven. The study on this hybrid filter exhibited that a thin top layer of electrospun PAN nanofibers (600 nm in diameter) could enhance the filtration efficiency without a considerable rise of the pressure drop.

Similarly, Wang *et al.* [91] fabricated an aerosol filter with a two-tier composite structure. This filter principally consists of a polyamide-66 (PA-66) nanofibrous top layer deposited on a conventional microfibrous nonwoven of PP. The amazing features of the electrospun nanofibrous toplayer including very small diameter (90–360 nm), high porosity and controllable coverage rate confer a high filtration efficiency (up to 99.9%), low pressure drop, easy cleanability and light weight to the hybrid air filter.

In another study by Patanaik *et al.* [48], the researchers developed a hybrid air filter, via incorporation of a polyethylene oxide (PEO) ENM between two nonwoven mats. The authors found out that with an increase in diameter of PEO nanofibers (85–125 nm), the filtration capacity is optimized and the pressure drop declines. As a finding of the research, it was stated that the cyclic compression applied during the working cycle determines the life span of the filter media. In this case, the hybrid filter exhibited a high resistance against cyclic compression. Consequently, due to a relatively less pressure drop, pore size remained constant and did not increase, *i.e.*, an improved filter efficiency and longevity compared to the single layer ENM/nonwoven filter [29].

As mentioned earlier, electrostatic attraction is a main mechanism of capturing of the sub-micron particles. Accordingly, it has been extensively employed in aerosol filters. For example, Yeom *et al.* [92] developed a nanocomposite ENM composed of nylon-6 nanofibers (73 nm in diameter) and boehmite nanoparticles. The nanoparticles were indeed electrostatic charging agents. As a result, with no significant alteration in the air flow resistance, a high capturing capacity for submicron aerosol was obtained. This optimum filtration efficiency could be attributed to increase of the surface potential of the nanofibers by, not only the boehmite nanoparticles, but also the electrospinning process itself [29,93].

### 4.2. Organic Gas Filtration

In addition to aerosols, organic gas pollutants are also detrimental to human health, comfort and productivity. Of them, the Volatile Organic Compounds (VOCs) are of the major pollutants daily inhaled [94,95]. Annually a considerable amount of VOCs is emitted into the air from industrial plants, vehicles, *etc.* VOCs, however, could be considered as indoor air pollutants as well. Inhalation of some of these compounds leads to sick building syndrome (SBS), including mucous membrane irritation, headache, fatigue [96–99], and even to cancer (e.g., formaldehyde, acrolein [100]). Accordingly, VOCs should be in an efficient way removed from outdoor and indoor environments [101]. Adsorption has been introduced as the most effective approach to reduce VOC levels and optimize air quality. Therefore, adsorbent materials, in the form of ENMs with a high surface area guarantying high removal efficiency at low-pressure drop, are an optimum candidate for this application.

Kim *et al.* [102] developed a nanocomposite ENM composed of fly ash incorporated PU nanofibers to remove VOCs from air. The fly ash particles (FAPs) as a byproduct of thermal plant were simply blended with PU solution and electrospun to form the composite nanofibers. This nanocomposite ENM was mechanically more durable than its neat counterpart. In addition, it showed an optimum potential in removal of different VOCs (chloroform, benzene, toluene, xylene, and styrene) through an absorption mechanism. The absorption capacity was proportional to the amount of FAPs in the fibers. Styrene was the most highly absorbed VOC. Among the nanocomposite ENMs, the one with 30 wt% FA exhibited the most optimum absorption capacity up to three times of the neat counterpart. Based on the obtained results, the FA/PU nanocomposite ENM could be considered as a suitable candidate for removal of VOCs from out/indoor environments.

### 4.3. Protective Clothing

Chemical warfare agents in the battlefields are usually in the form of aerosol or vapors. Hence, protective systems such as clothing and face masks are highly needed to safeguard the people from an eventual chemical or biological hazard [103].

**Figure 15.**
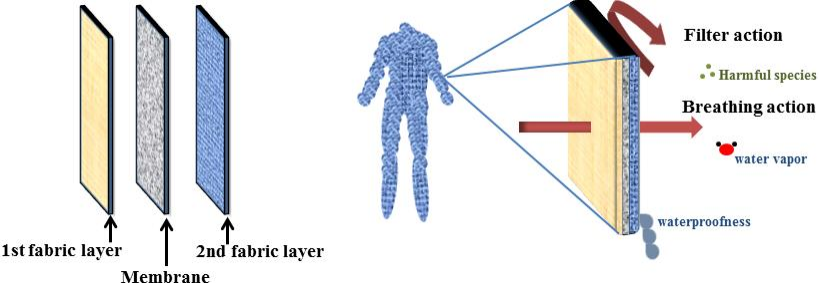
A breathable barrier membrane acting as protective clothing [104].

A fundamental requirement of suitable protective clothing is adaptability with the physiological conditions of the human body. Accordingly, while acting as a barrier against toxic and unwanted materials, such as aerosol particles, harmful vapors, and liquids, it should allow not only perspiration but also transportation of water vapor, thus creating a microclimate (Figure 15) [104].

ENMs possess the ability of selective permeation of moisture hence an optimum breathability also hindering of the chemical agents. Accordingly, they could be applied in protection against chemical and biological warfare contaminants as protective clothing [36,103–106]. Nanofibrous mats are extensively under investigation to find practicability in this area of air filtration domain.

A group at the National University of Singapore (NUS) [35] has recently explored the incorporation of nanoparticles such as MgO, TiO_2_, Al_2_O_3_ and other oxides into nanofibers for decontamination of a wide range of toxic gases including chemical contaminants, biological contaminants (viruses, bacteria), pesticides, *etc.* The protective clothing made on the basis of this nanocomposite ENMs has been thoroughly studied [103,107,108].

Sundarrajan and Ramakrishna [103], fabricated MgO nanoparticle incorporated polymer (poly(vinyl chloride)(PVC), poly(vinylidene fluoride-co-hexafluoropropylene) (PVDF copolymer), and polysulfone (PSU)) nanofibrous membranes for decontamination of the stimulant of nerve gas, paraoxon. The average fiber diameter of PVC, PVDF copolymer, and PSU membranes were found to be 555 ± 25 nm, 450 ± 18 nm, 500 ± 25 nm, respectively. In presence of MgO nanoparticles, for PVC and PVDF the diameter were reduced to 505 ± 13 nm, 415 ± 30 nm, respectively.

In this study, the incorporated nanoparticles were not fully exposed to the gas therefore a lower removal efficiency compared to the nanoparticles alone was obtained. However, the nanocomposite ENM of MgO/PSU (5% MgO) showed a two times more reactivity against chemical warfare agent stimulant, paraoxon, than the conventionally used charcoal.

To address the problem of less exposure of the incorporated nanoparticles, in a new study by Roso *et al.* [107], the nanoparticles were electrosprayed onto the electrospun nanofibers. In this study, depending on the quantity of surface residing nanoparticles, pressure drop declined.

Despite the advantages of such nanohybrid ENM structure, the nanoparticles present on the nanofibers could be leached out [36]. To stabilize the nanoparticles, recently, Sundarrajan *et al.* [109] benefited from a group of functional polymers, wherein the nanoparticles could interact with the hydroxyl groups on the surface. In this study, a blend solution of PET with cellulose acetate or cellulose was electrospun. Subsequently, metal oxide nanoparticles were deposited onto the nanofibers via liquid phase deposition (LPD) and electrospraying techniques. It was shown that when dip coating technique was employed, more nanoparticles were nucleated on the nanofibers with hydroxyl groups, *i.e.*, PET and cellulose or PET and cellulose acetate (CA). This group of nanocomposite ENMs shows a lower flow resistance also an optimized filtration performance thereby a high potential for being used as air filters (in hospitals, airplanes, industry, *etc.*), also protective clothing [36].

## 5. Future Perspective and Conclusions

Electrospun nanofibrous membranes (ENMs) are currently investigated extensively for environmental remediation. This high motivation stems from their unique structural characteristics including an extraordinary surface area also interconnected porosity. Based on such features; a remarkable selectivity also permeability thereby an optimum separation capacity are expected.

In air filtration, ENMs especially those reinforced by functional nanoparticles can optimally replace conventional glass fiber based filters. Outstripping the glass fiber filters is from a health point of view considerably important. Advantageous over High-efficiency particulate absorption (HEPA) filters, nanoparticular additives equip the ENM to the ability of removal of chemical contaminants. Also, catalyst nanoparticles exposed in a huge surface area of nanofibers enable decontamination of chemical and biological warfares in protective clothing and facemask applications. Therefore, benefits such as improved filtration efficiency, the protection duration and weight reduction are all met by the nanocomposite ENMs. One step further, catalytic also sensoric ENMs able to identify and neutralize hazardous chemical gases at the same time are highly demanded. Hence, they should be thoroughly investigated by the research labs in academy and industry in the future.

In contrary to the air filtration area, for water filtration, ENMs, due to some shortcomings, have not arisen significant interest for industrial investment. To move towards commercialization, drawbacks of low wettability, insufficient nanoscale selectivity, also mechanical weakness should be circumvented. Nanocomposite strategy could be the best solution. Addition of inorganic fillers also biological materials to the nanofiber templates confers not only an improved structural performance but also a tunable selectivity at the range of soluble also nanosubstances. When we incorporated ZrO_2_ and TiO_2_ nanoparticles to PES nanofibers, the resultant ENM exhibited a higher mechanical stability also wettability, thereby, a markedly better permeability. In case of immobilization of biological matters, such as proteins onto nanofibers, in addition to an enhanced surface chemical and mechanical properties, a smart interactive performance against environmental pollutants was obtained. A hydration induced conformational change of the protein led to capturing of tiny nanoparticles as small as 20 nm as well as biomolecules. Microorganisms are also assumed to be efficiently removed from water streams by such a biofunctionalized ENM. Accordingly, the application area would be extended to biomedicine in disinfection of biological liquids. This hypothesis is being evaluated in our lab. Besides the bioactivated systems, a photoswitchable nanocomposite nanofibrous membrane is also able to have a reversible wettability behavior upon UV and visible light illumination. Such a smart membrane can be used for oil/water separations, for example. This kind of switchable nanocomposite ENMs could be considered as multifunctional nanostructured membranes hence highly promising for environmental applications. Accordingly, an emphasis should be put on this group of advanced smart membranes for future researches in the area of environmental remediation.

On the whole, the next generation membranes for environmental applications would be based on cost effective and energy saving nanofibrous membranes. In this regard, nanocomposite ENMs would play a significant role when considering their smart, also tunable selectivity besides their extraordinary permeability and energy/cost efficiency. Recent advancements in nanocomposite nanofibrous membrane preparation, paved the way for a large number of filtration systems producing safe and clean environment. Undoubtedly, due to their ease of operation and greater efficiency, they will acquire an important role in the replacement of conventional membranes in the near future.
